# Transcriptomic epidemiology of smoking: the effect of smoking on gene expression in lymphocytes

**DOI:** 10.1186/1755-8794-3-29

**Published:** 2010-07-15

**Authors:** Jac C Charlesworth, Joanne E Curran, Matthew P Johnson, Harald HH Göring, Thomas D Dyer, Vincent P Diego, Jack W Kent, Michael C Mahaney, Laura Almasy, Jean W MacCluer, Eric K Moses, John Blangero

**Affiliations:** 1 Department of Genetics, Southwest Foundation for Biomedical Research, P.O. Box 760549, San Antonio, TX, USA; 2 Menzies Research Institute, Private Bag 23, Hobart, TAS, Australia

## Abstract

**Background:**

This investigation offers insights into system-wide pathological processes induced in response to cigarette smoke exposure by determining its influences at the gene expression level.

**Methods:**

We obtained genome-wide quantitative transcriptional profiles from 1,240 individuals from the San Antonio Family Heart Study, including 297 current smokers. Using lymphocyte samples, we identified 20,413 transcripts with significantly detectable expression levels, including both known and predicted genes. Correlation between smoking and gene expression levels was determined using a regression model that allows for residual genetic effects.

**Results:**

With a conservative false-discovery rate of 5% we identified 323 unique genes (342 transcripts) whose expression levels were significantly correlated with smoking behavior. These genes showed significant over-representation within a range of functional categories that correspond well with known smoking-related pathologies, including immune response, cell death, cancer, natural killer cell signaling and xenobiotic metabolism.

**Conclusions:**

Our results indicate that not only individual genes but entire networks of gene interaction are influenced by cigarette smoking. This is the largest *in vivo *transcriptomic epidemiological study of smoking to date and reveals the significant and comprehensive influence of cigarette smoke, as an environmental variable, on the expression of genes. The central importance of this manuscript is to provide a summary of the relationships between gene expression and smoking in this exceptionally large cross-sectional data set.

## Background

Tobacco use is responsible for more than 5 million deaths per year [1] and is the leading preventable cause of premature death worldwide. Smoking is known to have a major impact on human health, adversely affecting almost every organ. Exposure to cigarette smoke increases the risk of many diseases, including a wide range of cancers (from lung to pancreatic cancer), cardiovascular diseases (including atherosclerosis and coronary heart disease), a range of respiratory diseases (including chronic obstructive pulmonary disease and pneumonia), as well as various other adverse health effects such as increased risk of cataracts, infection and poor wound healing, and is generally detrimental to the overall health of individuals who smoke [2-8].

Investigating the influence of cigarette smoke exposure on health is a highly complex problem. The particulate and vapor phase of cigarette smoke contains in excess of 4,000 compounds, including five known human carcinogens and many toxic agents [9,10]. These toxins enter the bloodstream, via the pulmonary alveoli, and are distributed throughout the body. The widespread organ damage in active smokers reflects the systemic distribution of these compounds and the variety of cell types that are exposed. Studies of the effects of cigarette smoking have employed a variety of approaches to reduce the complexity of the problem, such as studying animal models or individual cell types *in vitro *that are exposed to 'standardized' measures of cigarette smoke, or to individual components of the particulate or vapor phases. However, no one model is able to capture the biological heterogeneity of the effects.

This study utilized large-scale genome-wide expression profiling as an alternative approach to determine the systemic influence of cigarette smoke, as an environmental exposure, on human physiology and health. Previous studies of gene expression as influenced by smoking have been seriously limited in size [11-21] with the largest of the *in vivo *studies including only 42 smokers and 43 non-smokers [15]. The small sample sizes and general lack of power have resulted in little concordance between these studies. Our hypothesis was that, given a sufficiently large set of related individuals, a stable and interpretable pattern of gene expression alterations attributable to cigarette smoke exposure may be obtained. In addition, a large and complex dataset allows for both the investigation of significant results at the individual gene level and provides the ability to determine elaborate networks of alteration. Studying these patterns of expression alteration in response to cigarette smoke exposure may provide the key to understanding the pathogenesis of many of the adverse health effects attributable to smoking and the interactions between them.

## Methods

### Ethics statement

All protocols were approved by the Institutional Review Board of the University of Texas Health Science Center at San Antonio. Participants gave informed consent and all investigation were conducted according to the principles expressed in the Declaration of Helsinki

### Study population

This investigation was conducted as part of the SAFHS, initiated in 1992 to investigate the genetics of cardiovascular disease and its risk factors in Mexican Americans [22]. Ascertainment occurred by way of adult probands selected at random, without regard to presence or absence of disease, from the Mexican American community in San Antonio, Texas. To ensure large, multigenerational pedigrees, probands had to have at least 6 age-eligible offspring and/or siblings living in San Antonio. All first, second, and third degree relatives of the proband and of the proband's spouse, aged 16 years or above, were eligible to participate in the study. More than 1,400 individuals from 42 extended families were recruited [22]. Reported family relationships were verified using the computer program PREST [23], based on autosomal genotype data, and corrections to the familial relationships were applied where required. Existing blood samples and phenotype data from the SAFHS were utilized in this investigation.

### Assessment of smoking status

Smoking status was assessed by structured interview conducted during the first clinic visit between 1991 and 1995, the same time point as the lymphocyte collection for the expression profiling. Data collected included current smoking status (smoker or non-smoker) as well as an estimate of cigarettes smoked per day, all by self report. No data were available on duration or former smoking status. In addition, serum cotinine levels were measured using a commercially available ELISA assay (BioQuant, San Diego, California). Serum for the cotinine assay was obtained during the first clinic visit but was only available for 981 of the 1,240 individuals with expression profiles.

### Expression profiling

The expression profiling methodology is described, in detail, in Göring *et al. *(2007) [24]. In brief, frozen lymphocyte samples were available from 1,240 individuals, collected during their first clinic visit between 1991 and 1995, after an overnight fast, in EDTA tubes. Lymphocytes were isolated from a 10 ml sample using Histopaque (Sigma Chemical Co., St. Louis, MO), following the suggested protocol of the manufacturer, washed, and stored in a freeze media in liquid nitrogen.

Total RNA was isolated using a modified procedure of the QIAGEN RNeasy^® ^96 protocol for isolation of total RNA from animal cells using spin technology (QIAGEN Inc., Valencia, CA), and a total of 500 ng total RNA dried down and stored at -20°C. Anti-sense RNA (aRNA) was synthesized, amplified and purified using the Ambion MessageAmp II Amplification Kit (Ambion, Austin, TX) following the Illumina Sentrix Array Matrix 96-well expression protocol (Illumina Inc., San Diego, CA). Synthesized cDNA samples were purified using QIAGEN's QIAquick 96 PCR purification supplementary protocol for spin technology (QIAGEN document QQ01.doc, October 2001). Biotin-16-UTP (Roche, Germany) labeled aRNA was synthesized using Ambion's proprietary MEGAscript^® ^in vitro transcription (IVT) technology and T7 RNA Polymerase. Purification of aRNA samples was performed using QIAGEN's RNeasy^® ^96 protocol for RNA cleanup using spin technology, and a total of 1.5 μg aRNA was dried and stored at -20°C prior to sample hybridization.

Hybridization of aRNA to Illumina^® ^Sentrix^® ^Human Whole Genome (WG-6) Series I BeadChips and subsequent washing, blocking and detecting were performed using Illumina's BeadChip 6 × 2 protocol, as described in Göring *et al. *[24]. Samples were scanned on the Illumina^® ^BeadArray™ 500GX Reader using Illumina^® ^BeadScan image data acquisition software (version 2.3.0.13). Illumina^® ^BeadStudio software (version 1.5.0.34) was used for preliminary data analysis, with a standard background normalization, to generate an output file for statistical analysis. In total we interrogated 47,289 unique transcripts: 22,151 probes (47%) are targeted at Reference Sequence (RefSeq) transcripts, and the remaining 25,128 probes (53%) correspond to other, generally less well characterized transcripts (including predicted genes) [24].

### Identification of expressed transcripts

In order to identify transcripts that exhibited sufficient quantitative expression in lymphocytes, the distribution of expression values for a given transcript was compared to the distribution of the expression values of the controls that are imbedded in each chip. For each transcript, we performed a χ^2 ^"tail" test of whether there was a significant excess of samples with values above the 95th percentile of the control null distribution. This test was used because it allows detection of even those transcripts that are clearly present above baseline levels in only a subset of individuals, while not being detectable above baseline levels in most individuals. Using a false discovery rate of 0.05, we identified 20,413 transcripts that exhibited significant expression by this criterion.

### Standardization of expression values

To minimize the influence of overall signal levels, which may reflect RNA quantity and quality rather than a true biological difference between individuals, abundance values of all 20,413 retained transcripts were first standardized by z-scoring within individuals (using decile percentage bins of transcripts, grouped by average log-transformed raw signals across individuals), followed by linear regression against individual-specific average log-transformed raw signal and its squared value. Lastly, for each transcript, we directly normalized these residual expression scores by employing an inverse Gaussian transformation across individuals, to ensure that the assumptions underlying variance components-based analyses were not violated. This conservative procedure results in normalized expression phenotypes that are comparable between individuals and across transcripts.

### Statistical Analyses

All statistical analyses on related individuals were performed using variance components-based methodology and software package SOLAR 4.1 [25]. To ensure that the assumption of a multivariate normal phenotypic distribution was not violated, we subjected all phenotypes to an exact inverse normalization procedure prior to analysis. We tested for association between smoking and gene expression levels using a regression model that allows for residual genetic effects, as implemented in SOLAR. In this approach, smoking was treated as a covariate for a given transcript's expression level. A likelihood ratio statistic was used to formally test the hypothesis that smoking was correlated with gene expression levels. This test was performed conditionally upon other covariate effects including those of sex, age, and their interactions. A false discovery rate approach [26] was utilized to deal with the major issue of multiple testing. We employed a rigorous FDR of 0.05 for all analyses.

We used combined discrete-continuous bivariate modeling analysis [27] to determine the environmental and genetic correlations between smoking (as a discrete trait) and expression of any given transcript (as a quantitative trait). Formal likelihood-based tests were used to test the difference of the genetic (ρ_g_) and environmental correlation (ρ_e_) from zero.

### Pathway and Networking Analysis

The 342 transcripts meeting the FDR of 0.05 criteria were analyzed using Ingenuity Pathways Analysis version 6.3 (Ingenuity^® ^Systems, http://www.ingenuity.com). There were 322 transcripts that mapped to known genes, including two cases where three significant transcripts corresponded to one gene (*GNLY *and *PID1*) and fifteen cases where there were two significant transcripts within a gene (*BCN2, C9ORF142, CDH23, CLEC10A, FLJ16686, LGR6, LMNA, LYPD2, MMP25, NCF4, SH2D3C, SNTB2, SSBP3, TCF7L2, TRA@ *and *ZAK*). In total there were 303 unique smoking correlated genes identified by IPA from the list of 342 significant transcripts. There were 20 transcripts that were unidentified and not included in the analyses, predominantly because their identifier had been retired or corresponded to a pseudogene (entrez gene IDs 28804, 80022, 255519) or hypothetical protein not yet described in the literature. All 323 unique identifiers (303 known and 20 unknown) are shown in Additional File 1.

The right-tailed Fisher's exact test was used to calculate a p-value determining the probability that each biological function and/or disease assigned to that dataset was due to chance alone. This p-value is calculated by comparing the number of user-imported genes in a given function or pathway relative to the total number of occurrences of these genes in all functional/pathway annotations stored in the knowledge base for the reference set. We used the entire set of 20,413 transcripts that were significantly detected in lymphocytes in our study [24] as the reference set for this investigation.

Our genes of interest were overlaid onto a global molecular network developed from literature reported connectivity recorded in the Ingenuity Pathways Knowledge Base, allowing the generation of gene networks; graphical representation of the molecular relationships between genes/gene products. All interactions between genes and other molecules in these networks are supported by peer reviewed publication.

### Accession Number

Raw expression values (of all transcripts on the microarray) and normalized expression values (of all 19,648 analyzed autosomal transcripts), along with information on sex and age are available under accession number E-TABM-305 on the ArrayExpress website http://www.ebi.ac.uk/arrayexpress/.

## Results

### Study Summary

For this study, transcriptional profiles were obtained from 1,240 Mexican American individuals from the San Antonio Family Heart Study (SAFHS). This dataset contained 1,154 individuals from 46 pedigrees and an additional 86 singletons. There were 734 females and 506 males in the sample, with a mean age of 39.3 years (SD = 16.7 years). Ages ranged between 16 and 94 years. For each sample, 47,289 transcripts were interrogated using the Sentrix Human-6 expression BeadChip supplied by Illumina (San Diego, CA). We were able to significantly detect 20,413 expressed transcripts in lymphocytes, 62.5% of these corresponding to known genes [24].

The prevalence of smoking in the dataset was 24%, with 297 current smokers. Using the genome-wide transcriptional profile dataset, we tested for correlations of gene expression in lymphocytes with a discrete measure of current smoking behavior, assessed by questionnaire. With a conservative false-discovery rate of 5%, corresponding to an observed nominal p-value of < 0.001, we identified 342 transcripts whose expression levels were significantly correlated with smoking, 110 (32.2%) with positive correlations and 232 (67.8%) negatively correlated. These 342 transcripts correspond to 323 unique genes. Details of this set of genes and the correlation of expression with smoking are provided in Additional File 1. Increasing the FDR to 10% increased the number of significant transcripts to 474, corresponding to an observed nominal p-value of < 0.0028.

### Validation of the phenotype

A quantitative measure of average cigarettes per day was available in this study, but was deemed less-reliable than the discrete trait, owing to bias introduced by self-report [28]. However, analyses using the quantitative measure did validate the set of smoking-correlated transcripts identified using the more conservative discrete trait, with a tetrachoric correlation of 0.905 ± 0.012 between transcripts significantly correlated with each measure at a 5% FDR.

In addition, plasma cotinine levels were available for a subset (79.1%) of the 1,240 studied individuals. Cotinine is a nicotine metabolite that is often used as a quantitative measure of cigarette smoking; however cotinine levels are subject to both genetic and environmental variation [29]. Using a plasma cotinine level of ≥20 for smoking and ≥300 for heavy smoking we only identified 17 individuals whose self-reported smoking status was clearly misclassified. The tetrachoric correlation between plasma cotinine levels and self-reported smoking status was extremely high (0.979 ± 0.007).

### Functional Annotation Analysis

In order to identify specific pathways and functional assignments involved in the response to smoking, we performed a series of formal pathway analyses. The 342 transcripts meeting the FDR of 0.05 criteria were analyzed using Ingenuity Pathways Analysis (IPA) version 6.3 (Ingenuity^® ^Systems, http://www.ingenuity.com). A total of 214 of the smoking-correlated genes included information on functions and/or canonical pathways from the published literature, which was used to identify overrepresentation of smoking correlated genes within known categories of functional assignments (such as immune response), and to develop hypotheses of gene action in the context of wider biological relationships. The most significant functional assignments included cell cytotoxicity (p = 3.7 × 10^-10^), immune response (p = 2.2 × 10^-7^), and tumorigenesis (p = 1.2 × 10^-6^). The most significant functional assignments are shown in Table 1, along with the p-value for the significance of each assignment and the total number of smoking correlated genes within each category. These are documented in more detail in Additional File 1, which includes the functional and canonical pathway assignments for each individual transcript within the dataset.

**Table 1:** The most highly significant functional assignments for the set of smoking correlated genes

Function Annotation	P-value	Number of genes
Cell cytotoxicity	3.7 × 10^-10^	17
Proliferation of cells	2.1 × 10^-9^	65
Activation of cells	1.7 × 10^-8^	31
Cell movement	2.9 × 10^-8^	35
Lysis of cells	1.2 × 10^-7^	11
Immune response	2.2 × 10^-7^	38
Mobilization of calcium	4.0 × 10^-7^	19
Adhesion of cells	7.8 × 10^-7^	29
Cell death	1.1 × 10^-6^	66
Tumorigenesis	1.2 × 10^-6^	72
Binding of cells	2.9 × 10^-6^	21
Viral elimination	4.3 × 10^-6^	3
Cancer	4.9 × 10^-6^	64
Inflammatory disorder	5.3 × 10^-6^	44
Cell growth	5.6 × 10^-6^	50
Cardiovascular disorder of lung	1.4 × 10^-4^	3
Perturbation of mitochondria	2.7 × 10^-4^	2
Inflammatory response	3.4 × 10^-4^	18
Asthma	2.5 × 10^-3^	9
Free radical scavenging	3.1 × 10^-3^	8

There were several highly significant functional categories involved in various aspects of cell death, including 17 genes involved in cell cytotoxicity (*CD38*, *CD300A*, *FASLG*, *FCGR3A*, *FCGR3B*, *GZMA*, *GZMB*, *KLRB1*, *KLRD1*, *KLRF1*, *KLRK1*, *PRF1*, *PTGDR*, *PTPN6*, *SLAMF7*, *SPN*, *TNFRSF8*; p = 3.7 × 10^-10^) and 11 in cell lysis (*ABCB1*, *CX3CR1*, *FASLG*, *FCGR3A*, *GNLY*, *GZMA*, *GZMB*, *KLRB1*, *KLRD1*, *KLRK1*, *PRF1*; p = 1.2 × 10^-7^) that were all significantly negatively correlated with smoking. In total there were 66 significant transcripts for genes involved in various aspects of cell death (p = 1.1 × 10^-6^), 47 negatively correlated and 19 positively correlated with smoking.

There were 38 smoking-correlated genes involved in immune response (p = 2.2 × 10^-7^), including 30 negatively correlated with smoking (*ADA*, *C3*, *CD38*, *CD247*, *CD300A*, *CST7*, *CTSC*, *CTSL1*, *CTSW*, *CX3CR1*, *FASLG*, *FCGR3A*, *FCGR3B*, *GFI1*, *GNLY*, *GZMA*, *GZMB*, *IL18RAP*, *IL2RB*, *ITGAX*, *KLRD1*, *KLRF1*, *KLRK1*, *PIK3CG*, *PRF1*, *SPN*, *SPON2*, *TGFBR3*, *TNFRSF14 *and *TRG@*) and eight positively correlated genes (*CLEC5A*, *EBI2*, *IGHE*, *MGST1*, *NCF4*, *RNASE2*, *SLAMF1 *and *TPSAB1*).

In addition, in the cell proliferation category there were 27 smoking-correlated genes related to lymphocyte proliferation, all but three of which were negatively correlated (p = 5.2 × 10^-7^). Twenty-one inflammatory response associated genes were significantly correlated with smoking (p = 3.4 × 10^-4^), including fourteen that were negatively correlated (*ADA*, *ADRB2*, *C3*, *CD38*, *CHST2*, *FASLG*, *GLNY*, *GZMA*, *IL18RAP*, *KLRG1*, *PIK3CG*, *PTGDR*, *PTPN6*, *SPON2 *and *TNFRSF14*) and seven that were positively correlated with smoking (*CLEC5A*, *IGHE*, *PLA2G7*, *RNASE2*, *S100A8*, *S100A12 *and *SERPINF1*).

There was evidence of overrepresentation of cancer related functional assignments, including 72 smoking-correlated genes that were associated with tumorigenesis (p = 1.2 × 10^-6^), 23 of which were positively correlated with smoking and 49 negatively correlated genes. There were also 64 genes associated with cancer (p = 4.9 × 10^-6^), 21 positively correlated with smoking (*ACOX2*, *AQP3*, *CANX*, *CLEC10A*, *CYP1B1*, *EBI2*, *EPB41L3*, *GPR177*, *IGL@*, *IL13RA1*, *LDHA*, *MGST1*, *MMP25*, *MTHFD2*, *NRG1*, *PAICS*, *S100A8*, *SERPINF1*, *TFDP1*, *UGCG *and *VCAN*) and 43 negatively correlated genes (*ABCB1*, *ADA*, *ADRB2*, *AKR1C3*, *ARIH2*, *AXIN1*, *C3*, *CACNA2D2*, *CD247*, *CDKN1C*, *CST7*, *CTSC*, *CTSL1*, *EBF4*, *FASLG*, *FCGR3A*, *GZMA*, *HMOX1*, *IL2RB*, *KLRK1*, *MT2A*, *NCAM1*, *ND3*, *NEURL*, *PALLD*, *PGLYRP2*, *PIK3CG*, *PODN*, *PPP2R2B*, *PRF1*, *PRSS23*, *PTGDS*, *PTPN6*, *RASSF1*, *RHOC*, *RRAS*, *SLC1A7*, *SSBP3*, *TGFBR3*, *TRA@*, *TRG@*, *TTC38 *and *UBE2C*).

In relation to respiratory-relevant pathologies, there were three genes associated with lung related cardiovascular disorder that were all negatively correlated with smoking (*FASLG*, *HMOX1*, *PRF1*; p = 1.4 × 10^-4^) and nine genes involved in asthma (p = 5.9 × 10^-4^) including six negatively correlated with smoking (*ADRB2*, *CX3CR1*, *GZMB*, *HMOX1*, *PTGDR *and *TNFRSF8*) and three positively correlated (*IGHE*, *NRG1 *and *PLA2G7*).

There was also some over-representation of free-radical related functional assignments, including two genes involved in mitochondrial perturbation (*GZMB *and *PRF1*; p = 2.7 × 10^-4^) and eight genes associated with free radical scavenging (p = 3.1 × 10^-3^); seven negatively correlated (*FASLG*, *GZMA*, *GZMB*, *HMOX1*, *PIK3CG*, *PRF1*, *RRAS*) and one (*IGHE*) positively correlated with cigarette smoking.

There were five significant canonical pathway categories of smoking correlated genes that also relate well to known smoking pathologies, shown in Table 2. The most significant of these canonical pathways assignments was for eleven genes involved in the natural killer cell signaling pathway, all of which were negatively correlated with smoking (*CD247*, *CD300A*, *FCGR3A*, *FCGR3B, KLRB1, KLRD1*, *KLRK1*, *PIK3CG*, *PTPN6*, *RRAS *and *SH2D1B*; p = 7.9 × 10^-7^).

**Table 2:** The most highly significant canonical pathway assignments for the set of smoking-correlated genes

Canonical pathway	P-value	Number of genes
Natural killer cell signaling	7.9 × 10^-7^	11
CTLA4 Signaling in cytotoxic T lymphocytes	1.2 × 10^-4^	8
SAPK/JNK signaling	5.7 × 10^-3^	6
Xenobiotic metabolism signaling	5.9 × 10^-3^	10
Metabolism of xenobiotics by cytochrome P450	9.6 × 10^-3^	4

There were ten genes involved in the xenobiotic metabolism signaling pathway in our dataset (p = 5.9 × 10^-3^), eight negatively correlated (*ABCB1*, *CHST2*, *CHST12*, *HMOX1*, *PIK3CG*, *PPP2R2B*, *PPP2R5A *and *RRAS*) and two that were positively correlated with smoking (*CYP1B1 *and *MGST1*). There were also four smoking-correlated genes in the related metabolism of xenobiotics by cytochrome P450 canonical pathway (p = 9.6 × 10^-3^), including three positively correlated (*CSGALNACT1*, *CYP1B1 *and *MGST1*) and one negatively correlated with smoking (*AKR1C3*). Cigarette smoke is a significant source of xenobiotics (chemicals foreign to the biological system) and these potentially damaging compounds are detoxified through such pathways.

### Network Analysis

**Figure 1:**
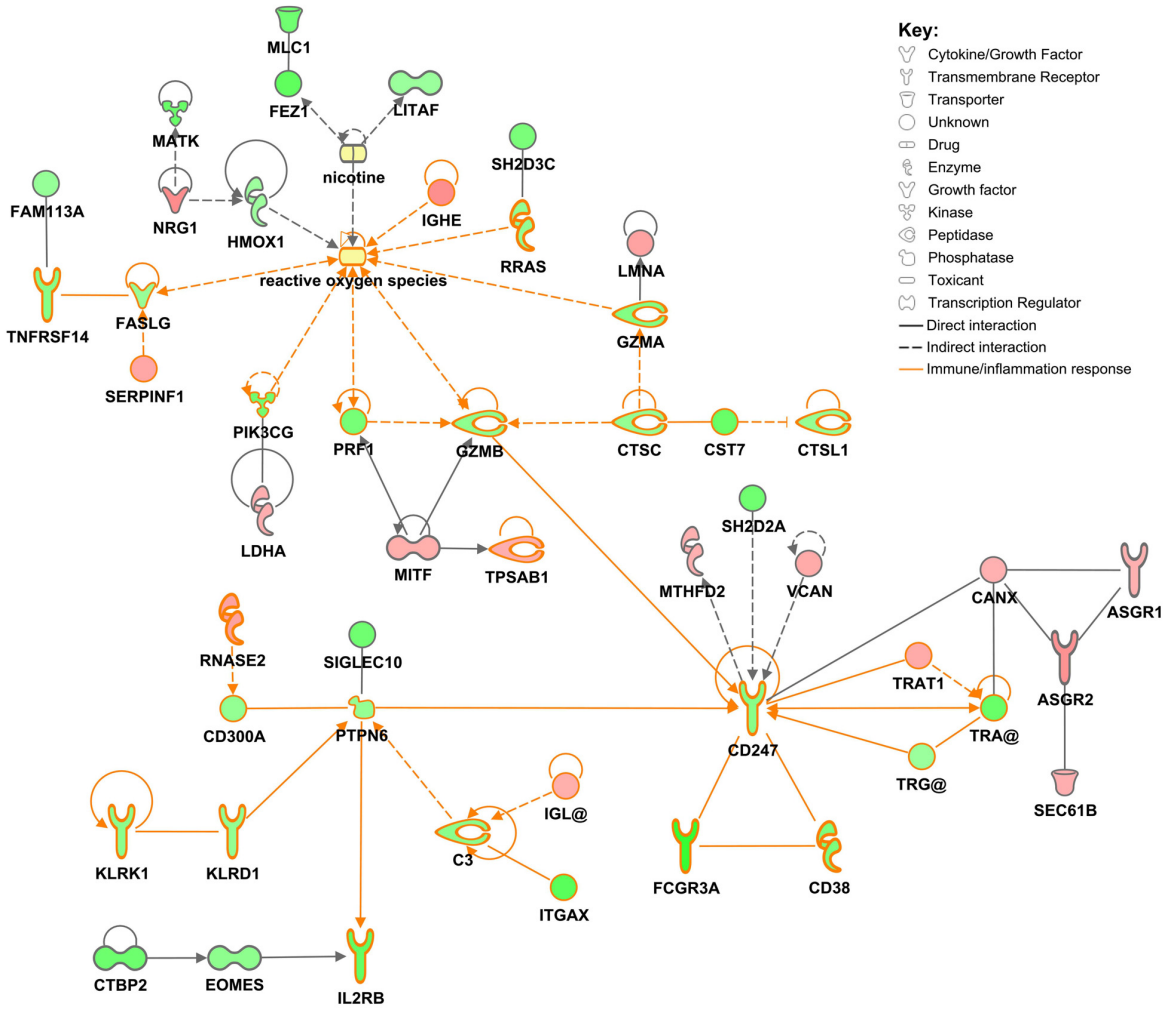
**A gene/gene product interaction network of smoking correlated genes**. Networks of gene/gene product interaction were generated using IPA (Ingenuity^® ^Systems, www.ingenuity.com). Genes or gene products are represented as nodes, and the biological relationship between two nodes is represented as an edge (line). All edges are supported by at least one published reference. Solid edges represent a direct relationship and dashed edges represent an indirect relationship. Node color represents the correlation of expression level with smoking, and the color intensity indicates the degree of correlation (red represents positive correlation while green represents negative correlation). The shape of each node represents the functional class of the gene product, as shown in the key. Yellow nodes indicate exogenous toxicants manually added to the network. Genes known to be involved in immune/inflammatory response are highlighted in orange.

There were 243 smoking-correlated genes with known connectivity information from published literature stored in the Ingenuity Knowledge Base. We used this published interaction information to determine whether our smoking-correlated expression signatures are tightly connected at the molecular level. This analysis is therefore restricted to interactions derived from the published literature and does not identify novel interactions between these genes. Network analysis was used to determine whether our smoking-correlated expression signatures are tightly connected at the molecular level, based only on these known interactions, by generating graphical representations of the interactions between genes and/or gene products in our dataset. Network analysis was used to connect 49 of the smoking correlated genes into a single network of gene/gene product interaction (Figure 1). This network includes a clear sub-network of 28 genes known to be involved in immune and inflammatory response (outlined in orange). Given their relevance to cigarette smoke exposure, we included the external toxicants nicotine and reactive oxygen species in the network.

### Genetic and Environmental Correlations of Expression with Smoking Behavior

Observed correlations between smoking behavior and a given gene's expression level may be due to the causal environmental effect of smoking on expression, the shared genetic determinants that jointly influence transcription level and the propensity to smoke, or a combination of these two influences. In order to assess the relative importance of environmental versus genetic sources of phenotypic covariation between expression levels and smoking status, we performed bivariate quantitative genetic analysis to decompose the observed phenotypic correlation of the 50 most significantly correlated transcripts. Because of our large pedigree-based study design, it is possible to directly estimate both the genetic and environmental correlations between expression levels and smoking status. Our results, documented in Additional File 2, clearly indicate that for all but one of the transcripts tested (98%) we saw no evidence for genetic correlation between smoking behavior and expression levels as would be expected if the observed correlation was the result of a genetic predisposition to smoking behavior. Given the strength of the estimated environmental component of covariation between smoking behavior and expression levels, our observed correlations most likely reflect the causal influence of smoking on transcription levels, which suggests that smoking is acting as a direct environmental mediator of transcription.

## Discussion

This study is the largest investigation of gene expression alterations in response to cigarette smoke exposure in human subjects *in vivo *to date. The results clearly reveal the broad influence of smoking, as an environmental influence, on the lymphocyte transcriptome. The results include a wide-ranging negative influence on the immune system, and strong involvement in a range of other relevant functional categories including cancer, cell death and xenobiotic metabolism. It is likely that this observed effect of smoking on transcription has larger implications for human disease risk, especially in relation to the increased risk of a wide variety of cancers throughout the body as a result of cigarette smoke exposure.

Peripheral lymphocytes appear to be an excellent surrogate tissue for investigating the effect of smoking on health by transcriptomic epidemiology. Not only are they one of the most readily and easily available tissues for gene expression analysis, they have also been shown to be a good surrogate for other tissue types in the case of environmental exposures, such as cigarette smoke, polyaromatic hydrocarbons and radiation [30-32]. The biological value of the lymphocyte as a surrogate for another more directly involved tissue (e.g., lung tissue) *does not *require that similar expression patterns be expressed between lymphocytes and the more focal tissue. It merely requires that, for a given gene, there is a phenotypic correlation between expression levels in the two tissues, which is much less restrictive than the requirements of similar absolute patterns of expression. Absolute levels are immaterial to our central hypothesis. It is highly likely that the regulatory machinery across tissue types is altered by mechanisms of attenuation or amplification which would lead to dramatically different absolute levels but still generate correlations between tissue types. If the attenuation/amplification mechanism has a linear component, then correlations will obligately result. Therefore, any regulatory feature that is shared across tissues will generate a correlation between tissues. Thus, a gene may be very highly expressed in one tissue and lowly expressed in a different tissue and still exhibit correlation between tissues. Such correlations are beginning to be demonstrated across a number of tissues [26].

Lymphocytes may be directly relevant for assessing the damaging effects of cigarette smoke. Thousands of cigarette smoke constituents are rapidly absorbed into the bloodstream, through the pulmonary alveoli, where they rapidly achieve systemic distribution. Oxidative damage and polycyclic hydrocarbon adducts have been readily detected, not only the respiratory epithelium and other first sites of exposure in cigarette smokers, but also in the blood and peripheral lymphocytes [33,34]. In addition, of the 47,289 transcripts interrogated in our study, we were able to significantly detect 20,413 expressed transcripts in lymphocytes, including both known and predicted genes [24]. This is an impressive level of diversity for any tissue, and allows the flexibility to identify signatures of gene expression correlated with a range of traits. Finally, since smoking is a major risk factor for a wide variety of cancers and diseases in a range of tissue types, it is important to understand its influence at the gene level outside of a selected cancer model.

All of the most significant functional groupings of smoking correlated genes identified in this study are directly relevant to well known smoking related disease processes. Cell death and proliferation, immune response, cancer, inflammatory disease and xenobiotic metabolism are all relevant groupings for smoking correlated genes, given known smoking related pathologies. However, the extent of these relevant groupings and the number of correlated genes whose expression is influenced by smoke exposure within each group is striking.

Various aspects of depressed immune function have been well documented in smokers [9,35-40]. We identified sets of smoking correlated genes corresponding to immune system components that fit with this profile of wide-spread immune alteration and suppression. For example, all 17 genes associated with cell cytotoxicity were negatively correlated with smoking; of the 29 genes involved in immune response (Table 1), the expression levels of 23 were negatively correlated with smoking; and of the sixteen genes involved in inflammatory response, twelve were negatively correlated with smoking. All eleven genes in the natural killer (NK) cell signaling pathway (Table 2), involved in cytotoxicity and cytokine secretion, were also negatively correlated with smoking. This wide-ranging negative influence on the immune system is one of the clearest pictures to emerge from our transcriptional profiling of smoking and gene expression. This comprehensive influence on immune related gene expression may go a long way towards explaining the processes behind the depressed immune system related pathologies exhibited by smokers.

As mentioned above, the expression levels of eleven genes in the natural killer (NK) cell signaling pathway were negatively correlated with smoking (*CD247*, *CD300A*, *FCGR3A*, *FCGR3B, KLRB1, KLRD1*, *KLRK1*, *PIK3CG*, *PTPN6*, *RRAS *and *SH2D1B*). NK cells are lymphocytes of the innate immune system involved in early defense against foreign cells and stressed autologous cells, and their cytotoxic activity is known to be decreased in smokers [36]. In addition, NK cell tumor immune surveillance was recently shown to be decreased in response to smoke exposure in a murine lung metastatic tumor model [41]. Our findings corroborate this negative influence of cigarette smoke on NK cell activity, and reveal some of the gene level alterations that may influence NK cells in smokers.

Another striking finding of this study was the over-representation of functional groupings relevant to cancer and cancer relevant processes such as cell death, proliferation and signaling. Cigarette smoking is a recognized risk factor for a wide variety of cancers, not only at the sites of contact such as the lungs and esophagus, but also throughout the body such as pancreatic, kidney, colon and bladder cancer. Correlations between expression of genes in lymphocytes and cigarette smoking in this study provide insight into the cancer relevant biological processes occurring throughout the body in response to smoke exposure, and hopefully serve to highlight the complexity of these processes.

We constructed a complex network based on published interaction between genes and/or gene products for 49 of the transcripts that were significantly correlated with smoking (Figure 1) including a clear sub-network of 28 genes known to be involved in immune and inflammatory response (outlined in orange). A portion of this network is also clustered around the exogenous toxicants nicotine and reactive oxygen species, with most of these genes involved in xenobiotic metabolism and free radical scavenging (*FASLG*, *GZMA*, *GZMB*, *HMOX1*, *IGHE*, *PIK3CG*, *PRF1 *and *RRAS*). The network displayed in Figure 1 clearly reveals the massive scale of influence that cigarette smoke, as an environmental variable, exerts over the lymphocyte transcriptome; however we are unable to determine which genes are directly influenced by smoke exposure, given that alteration of the expression level of one gene can alter the transcription of other genes in these networks. These interactions are made even more complex by the diversity of cigarette smoke components, and the variability of this constitution, in that we cannot identify which component(s) of cigarette smoke are exerting the most influence over gene expression. In addition, the network analysis is dependent on published connectivity information and as such cannot fully reflect the complexity of the interactions or reveal any novel connectivity. The literature reported interaction directionality is also of limited use in interpreting the empirical data, since our results reflect the state of the whole living organism. The observed correlations may reflect a large number of mediating factors including complex feedback loops that could easily be missed using classical in vitro cell-based models of cause and effect. However, this network does show that the genes whose expression is altered in response to cigarette smoke are tightly connected at the molecular level, and gives some indication of the pathways through which cigarette smoke influences known smoking related pathologies such as inflammation.

Although smoking has long-term adverse effects, cessation has some immediate, as well as long-term benefits, which may be due to a reversal of these transcriptomic alterations. Many of the negative health effects have been shown to be reversed or at least improved soon after cessation [4,41,42], for example elevation of NK activity (which is suppressed in smokers) is detectable within one month of smoking cessation [43]. This further supports the implication that at least part of the NK cell activity suppression is due to gene-level alterations in expression induced by smoke exposure, which may be reversed as the exposure is removed. However, a study comparing transcriptional profiles of 34 smokers, 23 non-smokers and 18 former smokers revealed that, while the majority of smoke exposure related gene expression alterations return to normal in former smokers, there were a set of transcripts that appeared to retain altered expression patterns two-years after smoking cessation [16]. Similarly, Beane *et al. *showed that 16% of the 175 differentially expressed genes identified in airway epithelial cells between smokers and non-smokers were irreversibly altered in former smokers [12]. Therefore, while it appears that the majority of gene expression alterations attributable to smoke exposure may be reversible, there may also be a subset of genes for which the expression changes are permanent or at least altered in the long term.

This study has some intrinsic limitations that should be noted. The study was conducted in a population of Mexican American individuals and it is difficult to determine what proportion of the expression changes would be replicated in other populations. While results obtained from within a subset of the population may not necessarily be relevant to all subsets of the population, in general it is highly likely that the majority of genes whose expression is altered in Mexican American individuals in response to smoke exposure are the same genes susceptible to alteration in other population groups. Transcriptional alterations are likely more robust to population differences than studies of individual genetic variants. However, a subset of the genes identified in this study may only be relevant to the Mexican American population. It is also important to note that, while not likely, it is possible that cigarette smoking is a surrogate for some other influence that is initiating the transcriptional alterations in this study.

## Conclusions

The results of this investigation offer insights into cigarette-smoke related pathological processes by determining its influences at the gene expression level. Never before has such a clear link between smoking and transcriptomics been revealed, and the scale at which exposure to cigarette smoke appears to influence the expression levels of our genes is sobering.

## Competing interests

The authors declare that they have no competing interests.

## Authors' contributions

MCM, LA, JWM, EKM and JB designed and instigated the study including the inception and maintenance of the San Antonio Family Heart Study, phenotype collection and expression profiling. JEC and MPJ conducted the laboratory analyses including the expression profiling. JCC, HHHG, TDD, VPD, JWK, MCM and JB cleaned and/or analyzed the family, phenotype and expression data. JCC conducted the bioinformatic analyses. JCC and JB wrote the manuscript. All authors read and approved the final manuscript.

## Pre-publication history

The pre-publication history for this paper can be accessed here:

http://www.biomedcentral.com/1755-8794/3/29/prepub

## Supplementary Material

Additional file 1 **Full details of the smoking-correlated genes significant at an FDR of 0.05**. Includes gene information, correlation of expression with smoking and the allocation of each gene into the functional assignments and canonical pathways from Ingenuity Pathways Analysis described in Tables 1 and 2. Click here for file

Additional file 2 **Bivariate analysis results for the fifty most significant smoking-correlated transcripts**. *Indicates there were multiple significant transcripts for this gene. Click here for file
